# Genetic Diversity and Association of EST-SSR and SCoT Markers with Rust Traits in Orchardgrass (*Dactylis glomerata* L.)

**DOI:** 10.3390/molecules21010066

**Published:** 2016-01-08

**Authors:** Haidong Yan, Yu Zhang, Bing Zeng, Guohua Yin, Xinquan Zhang, Yang Ji, Linkai Huang, Xiaomei Jiang, Xinchun Liu, Yan Peng, Xiao Ma, Yanhong Yan

**Affiliations:** 1 Department of Grassland Science, Animal Science and Technology College, Sichuan Agricultural University, Chengdu 611130, China; yanhaidong1991@163.com (H.Y.); zhangxq@sicau.edu.cn (X.Z.); jiangxiaomei830@163.com (X.J.); pengyanlee@163.com (Y.P.); maroar@126.com (X.M.); yanyanhong3588284@126.com (Y.Y.); 2 Institute of Agrifood Research and Technology (IRTA), Centre de Recerca en Agrigenòmica (CSIC-IRTA-UAB), Campus UAB–Edifici CRAG, Bellaterra, Cerdanyola del Vallès, Barcelona 08193, Spain; zhang.yu@cragenomica.es; 3 Department of Animal Science, Southwest University, Rongchang, Chongqing 402460, China; 4 Department of Crop, Soil, and Environmental Sciences, University of Arkansas, Fayetteville, AR 72704, USA; guohuayin1997@gmail.com; 5 Department of Grassland Science, Sichuan Animal Science Academy, Chengdu 610066, China; jiyang221@163.com; 6 Agricultural College, Sichuan Agricultural University, Chengdu 611130, China; ku0082005@126.com

**Keywords:** association analysis, EST-SSR, genetic diversity, orchardgrass, rust, SCoT

## Abstract

Orchardgrass (*Dactylis glomerata* L.), is a well-known perennial forage species; however, rust diseases have caused a noticeable reduction in the quality and production of orchardgrass. In this study, genetic diversity was assessed and the marker-trait associations for rust were examined using 18 EST-SSR and 21 SCoT markers in 75 orchardgrass accessions. A high level of genetic diversity was detected in orchardgrass with an average genetic diversity index of 0.369. For the EST-SSR and SCoT markers, 164 and 289 total bands were obtained, of which 148 (90.24%) and 272 (94.12%) were polymorphic, respectively. Results from an AMOVA analysis showed that more genetic variance existed within populations (87.57%) than among populations (12.43%). Using a parameter marker index, the efficiencies of the EST-SSR and SCoT markers were compared to show that SCoTs have higher marker efficiency (8.07) than EST-SSRs (4.82). The results of a UPGMA cluster analysis and a STRUCTURE analysis were both correlated with the geographic distribution of the orchardgrass accessions. Linkage disequilibrium analysis revealed an average r^2^ of 0.1627 across all band pairs, indicating a high extent of linkage disequilibrium in the material. An association analysis between the rust trait and 410 bands from the EST-SSR and SCoT markers using TASSEL software revealed 20 band panels were associated with the rust trait in both 2011 and 2012. The 20 bands obtained from association analysis could be used in breeding programs for lineage selection to prevent great losses of orchardgrass caused by rust, and provide valuable information for further association mapping using this collection of orchardgrass.

## 1. Introduction

Orchardgrass (*Dactylis glomerata* L.), a perennial and cool-season grassland species, is the fourth most economically significant forage grass genus grown worldwide [1]. Due to its high sugar and protein content, leaf yield, shade tolerance, and persistence, orchardgrass has been grown in East Asia, Europe, and North America for more than 100 years [2,3]. However, the majority of orchardgrass varieties are susceptible to rust disease (*Puccinia graminis* Pers.), which has caused a remarkable reduction in forage quality and production [4,5].

Recently, several studies have been conducted in order to control rust infection in orchardgrass. By comparing 126 orchardgrass accessions according to the phenotypic traits, Ittu and Kellner [6] discovered that orchardgrass from southern Europe showed the highest resistance to black rust, and they also demonstrated that orchardgrass originating from Denmark had lower rust resistance than accessions from Italy. Through phenotypic selection, Miller and Carlson [7] evaluated the rust resistance in orchardgrass based on both phenotypic performance and a polycross progeny test (phenotypic–genotypic method). Yan *et al*. [8] also screened 13 accessions with high disease resistance for a period of two years by evaluating the proportion of rust sorus on the leaves. All these studies depended on observing morphological characters; however, phenotypic traits are highly impacted by environmental conditions [9]. To address this question, genetic selection, a method that is seldom affected by the environment, is widely used to select desirable traits for plants [10].

For genetic selection, marker-assisted selection (MAS) has been developed to improve the efficiency of artificial selection among phenotypes by integrating molecular genetics in plant breeding [11,12]. The process of constructing linkage maps and analyzing quantitative trait loci (QTL) to identify genetic loci associated with traits is known as QTL mapping and is a critical first step for MAS. However, for QTL mapping, substantial time is needed to construct mapping populations and the intensive labor may limit the identification of specific traits using molecular markers. To remedy the limitations of this approach, association analysis, a method that can be used to analyze multiple traits without constructing mapping populations, has been widely used to identify molecular markers for traits using natural germplasm collections [13,14,15]. A number of association analyses with different molecular markers have been conducted to detect the loci related to rust resistance in common bean (*Phaseolus vulgaris* L.) [16], wheat (*Triticum aestivum* Linn.) [17], aspen (*Populus* L.) [18]. In numerous molecular markers, start codon targeted (SCoT) and expressed sequence tag-simple sequence repeats (EST-SSR) used in this study are two reliable markers [19,20], which have been successfully used to evaluate genetic diversity and population structure, and have assisted in selection for crop production [21,22,23,24].

At present, no study has focused on association analyses between rust traits and molecular markers in orchardgrass. In this study, our objective is to (1) identify the degree of genetic diversity and establish the relationships between different accessions of orchardgrass using EST-SSR and SCoT markers and (2) explore associations between EST-SSR and SCoT and rust traits. We hope that our data will contribute to an understanding of the inheritance of the rust trait and lay the basis for MAS in orchardgrass.

## 2. Results

### 2.1. EST-SSR and SCoT Analysis

Six accessions of orchardgrass, including three rust-resistant (1473; 947; 02-115) and three rust-susceptible (PI111536; PI595173; PI578634) samples, were selected to screen 100 pairs of EST-SSR primers. In total, 18 of EST-SSR primers generated robust discernible bands (Table S1). A total of 164 reliable bands were identified, of which 148 were polymorphic (90.24%), with an average value of 8.22 polymorphic bands per primer and a range of five to 12 bands (Table S2). These results indicate that EST-SSR primers have high amplification efficiency and are reliable in the discovery of polymorphisms.

Forty-eight SCoT primers were also tested using total DNA samples from three rust-resistant and three rust-susceptible samples to select primers. All 48 primers generated polymorphic PCR amplification products; however, 21 of the primers that could produce clear and reproducible bands were selected for further study (Table S3). In total, 289 bands were generated and 272 (94.12%) were polymorphic, with an average value of 12.95 polymorphic bands per primers and a range of seven to 20 bands (Table S4). Therefore, SCoT markers displayed amplification efficiency.

Based on simple matching coefficients, data from the two assembled markers (EST-SSR and SCoT) were used to detect the genetic similarity coefficient (GS) between pairs of orchardgrass samples. The analysis with NTsys-pc V2.1 showed a GS value from 0.532 to 0.832, with an average value of 0.638. Shannon’s information index of diversity based on EST-SSRs and SCoTs was 0.538, which indicated a rich genetic variance in the 75 orchardgrass accessions. Nei’s gene diversity index was 0.369.

The AMOVA of the distance matrix for all accessions permitted a partitioning of the overall variations into two levels: among populations and within populations. The results showed that most of the genetic variations existed within populations. The proportion of variations attributed within populations was 87.57%, and the remainder of variations (12.43%) occurred among populations. As a result, obvious genetic differentiation existed within the orchardgrass populations.

### 2.2. Markers Efficiency Analysis

The efficiencies of the EST-SSRs and SCoTs were compared using a parameter MI for 75 orchardgrass accessions (Table 1). There was little inconsistency between the Ibav indexes for EST-SSRs and SCoTs, and their values were 0.59 and 0.62, respectively. However, the EMR index of SCoTs (12.95) was larger than that of EST-SSRs (8.22). The MI calculation indicated a distinctive and highly efficient nature of the SCoTs, with the MI for this marker an order of magnitude (8.07) higher than MI in the EST-SSRs (4.82).

**Table 1:** Comparison of usefulness between EST-SSR and SCoT markers for 75 orchardgrass accessions.

Items	EST-SSRs	SCoTs
Number of primers	18	21
Number of total loci	164	289
Number of average loci per primers	9.11	13.76
Percentage of polymorphic bands	0.90	0.94
Average band informativeness (Ibav)	0.59	0.62
Effective multiplex ratio (EMR)	8.22	12.95
Mark index (MI)	4.82	8.07

### 2.3. Cluster Analysis

**Figure 1:**
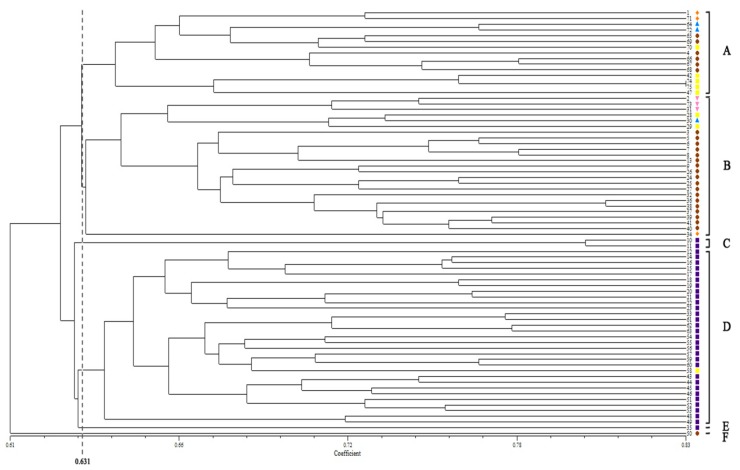
The UPGMA dendrogram of 75 orchardgrass accessions. The symbols represent the geographic groups in cluster tree as ◆ (orange) accessions from North America, ● (brown) accessions from Asia, ■ (purple) accessions from Europe, ■ (yellow) accessions from Africa, ▲ (blue) accessions from South America, and ▼ (pink) accessions from Oceania. A–F represent the 75 orchardgrass samples could be clearly divided into six groups by the UPGMA dendrogram when the genetic coefficient was approximately 0.631.

The 75 orchardgrass samples could be clearly divided into six groups (A–F) by the UPGMA dendrogram based on Dice GS when the genetic coefficient was approximately 0.631 (Figure 1). The results from the UPGMA were relative to the geographical distribution of the orchardgrass. For example, Group A contained 15 accessions that were predominantly from Asia and Africa; Group B consisted of 25 accessions that were nearly all from Asia; Thirty-five accessions from Group C, D, E, and F were nearly all from Europe, except two from Africa and Asia, respectively.

### 2.4. Population Structure Analysis

**Figure 2:**
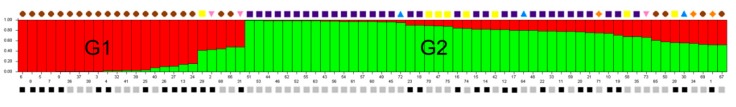
Four subgroups inferred from STRUCTURE analysis. The vertical coordinate of each subgroup means the membership coefficients for each accessions; the digits of the horizontal coordinate represent the accessions corresponding to Table S5; Red zone: G1, Green zone: G2, Blue zone: G3, and Yellow zone: G4; The colored samples upon the figure indicate the geographic distribution information of accessions. ◆ (orange) accessions from North America, ● (brown) accessions from Asia, ■ (purple) accessions from Europe, ■ (yellow) accessions from Africa, ▲ (blue) accessions from South America, and ▼ (pink) accessions from Oceania; The simples below the figure indicate the rust trait information of accessions. ■ (black) accessions with resistant (HR and R) traits, and ■ (grey) accessions with susceptible traits (HS and S).

Population structure of the 75 accessions was estimated using STRUCTURE V2.3.4 software (version 2.3.4, Pritchard lab, Stanford University, Palo Alto, CA, USA) based on 18 EST-SSR markers and 21 SCoT markers. The maximum likelihood and ΔK were used to calculate the number of subpopulations (K), with accessions falling into two subgroups. With a membership probability threshold of 0.60, 19 accessions were assigned to group 1 (G1), 44 accessions to group 2 (G2) and 12 accessions were retained in the AD. With the maximum membership probability, 24 accessions were assigned to G1, 51 accessions to G2. The relationship between the geographic distribution of orchardgrass accessions from Asia, Europe, North America, South America, Oceania, Africa, and the subgroups derived from the STRUCTURE analysis was further analyzed. G1 comprised 21 accessions from Asia, two from Oceania and one from Africa. Most of the accessions (33) from G2 came from Europe (Figure 2). Among these 75 accessions, most in Asia and Europe could be distinguished using STRUCTURE (Figure 2), indicating that the population structure assigned by the STRUCTURE analysis might be correlated with the geographic distribution of these orchardgrass accessions.

### 2.5. LD Analysis

Eighteen EST-SSRs and 21 SCoT markers with unknown chromosome information were used to assess the extent of LD in the 75 orchardgrass accessions. Across all 410 bands amplified by 39 markers, 83,845 pairs of bands were detected, of which 1927 pairs of bands (2.30%) were considered in LD at *p* < 0.01, and 1769 bands pairs (2.11%) were found at r^2^ > 0.1 and *p* < 0.01. The values of r^2^ in association between the 1927 pairs of bands were calculated, and the data ranged from 0.0000 to 1.0000, with an average r^2^ of 0.1627, indicating a high extent of LD existing in these bands.

### 2.6. Phenotype Analysis

The phenotype data for the survival of the individuals in the field for two years were listed in Table S5. The number of rust resistant and sensitive accessions from Asia and Europe were approximately equal: in Asia, 11 accessions appeared HR or R, and 14 accessions were HS or S; in Europe, 13 accessions were HR or R, while 20 accessions were HS or S. However, the number of rust resistant and sensitive accessions in Africa is imbalanced, for that six rust sensitive accessions in Africa were dominant. The orchardgrass accessions originated from six continents that had the rust trait and sensitive accessions, which indicated these materials were reliable for our further association analyses (Table 2).

**Table 2:** The number of accessions for trait information about orchardgrass that distribute in 6 continents.

Continent	Trait	The Number of Accessions
Asia	resistance	11
	susceptibility	14
Europe	resistance	13
	susceptibility	20
North America	resistance	2
	susceptibility	1
South America	resistance	1
	susceptibility	2
Oceania	resistance	2
	susceptibility	1
Africa	resistance	2
	susceptibility	6

### 2.7. Association Analysis

The association analysis between EST-SSR and SCoT markers and important phenotypic traits was performed using the TASSEL GLM to find the associated tags and allelic variation. Among the 410 bands in the 18 EST-SSR and 21 SCoT markers, there were 75 bands that were highly significantly (*p* < 0.01) associated with the rust trait for two years. In 2011, 39 gene-trait association bands were found across 75 accessions of orchardgrass under rust infection, while 36 bands were identified to be associated with the trait in 2012 (Table 3). Interestedly, 20 bands (Table 4) were found to be associated with the rust trait in both 2011 and 2012. Additionally, the explanation of all these bands ranged from 7.98% to 12.53%, and the top three bands with the highest explanation were 156 (12.53%), 321 (12.41%), and 157 (12.37%) in 2012 (Table 3).

**Table 3:** Significance test between alleles and phenotypic variation for association analysis in orchardgrass (*p* < 0.01).

Location	Year	Alleles	*p* Value (<0.01)	r^2^ Value
SCo2012-680 bp	2012	156	0.0010	0.1253
SCoT37-280 bp	2012	321	0.0011	0.1241
SCo2012-600 bp	2012	157	0.0011	0.1237
SCo20126-720 bp	2011	268	0.0012	0.1217
SCo2012-680 bp	2011	156	0.0013	0.1209
SCoT41-1000 bp	2012	368	0.0013	0.1205
SCoT44-420 bp	2011	408	0.0015	0.1179
SCoT37-1000 bp	2011	311	0.0015	0.1172
EST-SSRH41-42-280 bp	2012	22	0.0016	0.1168
SCoT44-390 bp	2011	409	0.0015	0.1167
SCoT37-850 bp	2012	312	0.0016	0.1162
SCo20110-1000 bp	2012	207	0.0017	0.1153
SCo20110-580 bp	2011	212	0.0017	0.115
SCo20116-260 bp	2011	250	0.0017	0.1144
EST-SSRH41-42-280 bp	2011	22	0.0019	0.1131
SCo20114-580 bp	2011	225	0.0019	0.113
SCo20125-1000 bp	2011	251	0.0019	0.113
SCoT37-350 bp	2012	319	0.0019	0.1129
SCo20110-340 bp	2012	220	0.0021	0.1115
EST-SSRH59-60-275 bp	2012	53	0.0021	0.1114
SCo20126-830 bp	2011	266	0.0021	0.1107
SCoT37-850 bp	2011	312	0.0021	0.1107
SCo20110-200 bp	2011	223	0.0021	0.1106
SCoT42-300 bp	2012	395	0.0022	0.11
SCoT42-860 bp	2012	383	0.0023	0.109
SCoT44-750 bp	2011	402	0.0023	0.1089
SCo20125-850 bp	2011	252	0.0023	0.1087
SCoT34-250 bp	2012	292	0.0025	0.1079
EST-SSRH59-60-275 bp	2011	53	0.0025	0.1067
SCo20127-750 bp	2011	273	0.0026	0.1063
SCo20110-340 bp	2011	220	0.0026	0.1062
SCoT8-260 bp	2012	201	0.0027	0.1056
SCo20116-410 bp	2011	248	0.0027	0.1052
SCoT8-260 bp	2011	201	0.0028	0.105
EST-SSRH39-40-175 bp	2012	16	0.0028	0.1049
SCoT36-350 bp	2012	305	0.0030	0.1035
SCoT44-750 bp	2012	402	0.0031	0.1034
SCo20110-1000 bp	2011	207	0.0031	0.1026
EST-SSRH61-62-175 bp	2011	63	0.0038	0.0985
SCoT42-320 bp	2012	394	0.0041	0.0977
EST-SSRH47-48-85 bp	2012	34	0.0041	0.0976
SCoT42-320 bp	2011	394	0.0045	0.0951
SCoT37-1500 bp	2012	310	0.0046	0.0951
SCoT40-230 bp	2011	364	0.0046	0.0947
SCo20126-830 bp	2012	266	0.0047	0.0946
SCo20125-200 bp	2012	264	0.0048	0.0944
SCo2012-720 bp	2011	155	0.0048	0.094
SCoT42-1800 bp	2011	380	0.0051	0.0929
SCo20127-350 bp	2012	280	0.0053	0.0923
EST-SSRH77-78-140 bp	2012	119	0.0058	0.0904
SCo20125-220 bp	2012	263	0.0060	0.0898
SCoT6-1100 bp	2012	180	0.0060	0.0897
SCoT39-500 bp	2011	343	0.0060	0.0896
SCo20126-680 bp	2011	269	0.0061	0.0892
SCo20125-220 bp	2011	263	0.0062	0.0888
SCoT6-1100 bp	2011	180	0.0063	0.0886
SCoT34-450 bp	2011	285	0.0064	0.0882
SCoT40-250 bp	2011	363	0.0065	0.0879
SCoT35-500 bp	2012	296	0.0067	0.0876
SCoT39-100 bp	2012	351	0.0069	0.087
SCoT34-360 bp	2011	287	0.0069	0.0867
EST-SSRH77-78-140 bp	2011	119	0.0070	0.0864
SCo20126-400 bp	2011	271	0.0075	0.0849
SCo2012-470 bp	2012	159	0.0077	0.0847
SCoT41-700 bp	2011	371	0.0076	0.0846
SCoT39-100 bp	2011	351	0.0078	0.0842
SCo20126-720 bp	2012	268	0.0082	0.0834
SCoT36-290 bp	2012	307	0.0083	0.0832
SCoT42-860 bp	2011	383	0.0082	0.083
SCo2012-470 bp	2011	159	0.0083	0.0829
SCoT44-390 bp	2012	409	0.0085	0.0827
SCo20125-850 bp	2012	252	0.0085	0.0826
SCoT5-300 bp	2012	178	0.0086	0.0824
EST-SSRH47-48-230 bp	2012	29	0.0095	0.0805
SCo20126-400 bp	2012	271	0.0098	0.0798

**Table 4:** The information about alleles that are associated with the rust resistance trait for orchardgrass.

Location	Year	Alleles	*p* Value (<0.01)	r^2^ Value	Mean r^2^ Value
SCoT44-390 bp	2011	409	0.0015	0.1167	0.0997
SCoT44-390 bp	2012	409	0.0085	0.0827	
SCoT44-750 bp	2011	402	0.0023	0.1089	0.1062
SCoT44-750 bp	2012	402	0.0031	0.1034	
SCoT42-320 bp	2012	394	0.0041	0.0977	0.0964
SCoT42-320 bp	2011	394	0.0045	0.0951	
SCoT42-860 bp	2012	383	0.0023	0.1090	0.0960
SCoT42-860 bp	2011	383	0.0082	0.0830	
SCoT39-100 bp	2012	351	0.0069	0.0870	0.0856
SCoT39-100 bp	2011	351	0.0078	0.0842	
SCoT37-850 bp	2012	312	0.0016	0.1162	0.1135
SCoT37-850 bp	2011	312	0.0021	0.1107	
SCo20126-400 bp	2011	271	0.0075	0.0849	0.0824
SCo20126-400 bp	2012	271	0.0098	0.0798	
SCo20126-720 bp	2011	268	0.0012	0.1217	0.1026
SCo20126-720 bp	2012	268	0.0082	0.0834	
SCo20126-830 bp	2011	266	0.0021	0.1107	0.1027
SCo20126-830 bp	2012	266	0.0047	0.0946	
SCo20125-220 bp	2012	263	0.0060	0.0898	0.0893
SCo20125-220 bp	2011	263	0.0062	0.0888	
SCo20125-850 bp	2011	252	0.0023	0.1087	0.0957
SCo20125-850 bp	2012	252	0.0085	0.0826	
SCo20110-340 bp	2012	220	0.0021	0.1115	0.1089
SCo20110-340 bp	2011	220	0.0026	0.1062	
SCo20110-1000 bp	2012	207	0.0017	0.1153	0.1090
SCo20110-1000 bp	2011	207	0.0031	0.1026	
SCoT8-260 bp	2012	201	0.0027	0.1056	0.1053
SCoT8-260 bp	2011	201	0.0028	0.1050	
SCoT6-1100 bp	2012	180	0.0060	0.0897	0.0892
SCoT6-1100 bp	2011	180	0.0063	0.0886	
SCo2012-470 bp	2012	159	0.0077	0.0847	0.0838
SCo2012-470 bp	2011	159	0.0083	0.0829	
SCo2012-680 bp	2012	156	0.0010	0.1253	0.1231
SCo2012-680 bp	2011	156	0.0013	0.1209	
EST-SSRH77-78-140 bp	2012	119	0.0058	0.0904	0.0884
EST-SSRH77-78-140 bp	2011	119	0.0070	0.0864	
EST-SSRH59-60-275 bp	2012	53	0.0021	0.1114	0.1091
EST-SSRH59-60-275 bp	2011	53	0.0025	0.1067	
EST-SSRH41-42-280 bp	2012	22	0.0016	0.1168	0.1150
EST-SSRH41-42-280 bp	2011	22	0.0019	0.1131	

## 3. Discussion

MI, as an efficiency indicator, is a convenient estimate for marker efficiency [25]. The MI (8.07) for SCoTs was larger than that in EST-SSRs (4.82), indicating a higher efficiency in the SCoTs (Table 1). This is due to the EMR component, and the MI was higher in the SCoTs (12.95) than the EST-SSRs (8.22), when the Ibav of these two markers were similar (0.59 for EST-SSR *vs*. 0.62 for SCoT; Table 1). This result corroborates studies conducted on tetraploid potato (*Solanum tuberosum*), in which the MI value of the SCoTs was shown to be higher than that of the EST-SSRs [26], Persian oak (*Quercus brantii* Lindl.), in which the SCoTs were more informative than inter-retrotransposon amplified polymorphism (IRAP) and inter-simple-sequence-repeat (ISSR) markers for the assessment of diversity [27], and mango (*Mangifera indica* L.), in which the SCoT assay better represents the actual relationships than ISSR analysis [28]. Additionally, the high MI of the SCoT marker from its highly efficacious multiplex ration may be proper for evaluating genetic diversity in breeding populations [29,30] or for fingerprinting [31].

Population structure is a significant factor that strongly influences association analyses. The unequal distribution of bands within groups can cause spurious associations [32]. In this study, 75 orchardgrass accessions can be classified into two groups (Figure 2) relating to geographical origin in the STRUCTURE analysis. The UPGMA cluster analysis also confirmed the geographical division of the groups (Figure 1). Compared with the STRUCTURE with the maximum membership probability and UPGMA results, the accessions in G1 and Group B predominately came from Asia, G2 and Group D contained accessions that mostly originated from Europe. These results once again indicated that the orchardgrass population was positively correlated with geographic distribution. It is essential for population-based methods to separate accessions from mixed populations into several unstructured subpopulations and to analyze the association between phenotypes and bands in homogeneous subpopulations [33,34,35]. The spurious associations are constantly considered when the accessions with particular phenotypes are biased to specific subpopulations [35,36,37]. In this study, with the maximum membership probability, accessions associated with resistant (HR and R) and susceptible traits (HS and S) both remained in most subpopulations (G1 and G2) (Figure 2), indicating that this orchardgrass population was applicable to association analysis.

Molecular markers associated with traits have been applied to a large number of common crops such as maize (*Zea mays*) [38], barley (*Hordeum vulgare* L.) [39], soybean (*Glycine max*) [35], and peanut (*Arachis hypogaea* L.) [40]. However, no study has been conducted on orchardgrass. The contribution of 20 band panels that appeared to be significantly (*p* < 0.01) associated with the trait explained 8.24%–12.31% in both 2011 and 2012 (Table 4). This indicates that the genetic effects of quantitative trait genes controlling the rust trait of orchardgrass may be unvaried in magnitude, which further confirmed the detection of major bands controlling the rust trait for orchardgrass. Markers associated with rust analysis have also been applied in other forage grasses. For example, Muylle *et al*. [41] detected two clusters of AFLP markers in perennial ryegrass (*Lolium perenne*) and one cluster mapped to linkage group two, a known genomic region containing crown rust resistance genes (6.1% explanation variance), while another cluster that was unlinked to the cluster on linkage group two was a novel genomic region of major effect that explained 27.7% of LD. Due to a lack of association mapping, we could not identify the location on specific chromosomes for the detected bands; however, these 20 band panels could provide valuable guidance on association analysis for marker-related rust traits and could be selected in breeding to potentially avoid great commercial and environmental loss of orchardgrass caused by rust.

## 4. Materials and Methods

### 4.1. Experimental Materials and the Rust Trait

A total of 75 orchardgrass accessions, with resistance of disease (R), high level of resistance of disease (HR), susceptibility of disease (S), and high susceptibility of disease (HS), as evaluated by Yan *et al*. [8], were chosen as experimental materials, and 31 out of the 75 accessions were scored as having HR or R, while the others (44 out of 75) were scored as having S or HS in 2011 or 2012 (Table S5). The HR, R, S, and HS groups were assigned to 6, 5, 2, 1, respectively, to be the phenotypic value for next association analysis (Table S5). The germplasm of orchardgrass used in this experiment consisted of 75 accessions that originated from Asia, Africa, Europe, North America, South America, and Oceania. For each accession, 20 individuals were randomly collected, and 0.5 g of clean, young leaves were selected per plant in 2011 for further DNA extraction.

### 4.2. DNA Extraction and EST-SSR and SCoT Amplification

The total genomic DNA was extracted using the DNeasy Plant Mini Kit (Qiagen, New York, NY, USA). The quantity and quality of the DNA was inspected using 0.8% gel electrophoresis. The quantified DNA was stored at –20 °C and was diluted to 20 μg/µL before usage.

EST-SSR primers were synthesized at Shanghai Sangon Biological Engineering Technology and Service Company (Shanghai, China). Eighteen primers that could amplify clear bands from 100 EST-SSR primers were selected for further analysis (Table S1). PCR amplification was performed in a 15 μL reaction system composed of: 1 μL of 20 ng/μL DNA, 7.5 μL of mixture (10× reaction buffer, 2.0 mM Mg^2+^, 0.6 mM of each dNTPs), 2 μL of 10 pmol/μL forward and reverse primers, 0.4 μL of 2.5 U/μL Golden DNA Polymerase (Tiangen Biotech, Beijing, China), and 4.1 μL of ddH2O. The PCR amplification program was as follows: initial denaturation at 94 °C for 10 min, 35 cycles of denaturation at 94 °C for 30 s, annealing at 59 °C for 30 s, extension at 72 °C for 30 s with a final extension at 72 °C for 5 min, and storage at 4 °C. The EST-SSR PCR fragments were separated on a 6% denatured polyacrylamide gel (acrylamide: bis-acrylamide 19:1, 1 × TBE). The gel was stained with an AgNO_3_ solution and then was photographed using the Gel Doc XR system (Bio-Rad, Hercules, CA, USA).

SCoT primers were also synthesized at the Shanghai Sangon Biological Engineering Technology and Service Company (Shanghai, China). After initial selection, 21 out of 48 primers that produced clear bands were used for further analysis (Table S2). The PCR reaction system was same as used for EST-SSR detection, except that 1.5 μL of 10 pmol/μL forward and reverse primers and 4.6 μL of ddH2O were added to the reaction system. The PCR amplification program was as follows: initial denaturation at 94 °C for 3 min, 36 cycles of denaturation at 94 °C for 50 s, annealing at 50 °C for 1 min, extension at 72 °C for 2 min with a final extension at 72 °C for 5 min, and storage at 4 °C. The detection and photography of PCR fragments was the same as with the EST-SSR primers.

### 4.3. Genetic Diversity and Cluster Analysis

The amplified fragments of each EST-SSR and SCoT marker were scored as “1” for presence and “0” for absence. The diversity parameters, total number of bands, number of polymorphic bands, percentage of polymorphic bands, Nei’s [42] gene diversity index and Shannon’s information index were estimated using POPGENE v.1.32 [43] and Excel 2007. A dendrogram was constructed in NTSYS-pc (version 2.1, Crop and soil science, Michigan State University, Lansing, MI, USA) using the un-weighted pair-group mean algorithm (UPGMA) cluster [44]. AMOVA (v.1.55, University of Geneva, Geneva, Switzerland) was employed to reveal the genetic variation among the groups and within populations [45]. The data input to POPGENE and AMOVA was produced using DCFA v.1.1 [46].

### 4.4. Markers Efficiency Analysis

Marker index (MI) was used to assess the efficiency of EST-SSR and SCoT markers in 75 orchardgrass accessions. MI is the average band informativeness (Ibav) for the polymorphic markers and the effective multiplex ratio (EMR) [47]. Ibav is defined as follows:

(1) $$\text{Ibav}=1/\text{n}\sum^{\text{​}}1-\left(2|0.5-\text{pi}|\right)$$

where pi is the proportion of the ith amplification site and n represents the total number of amplification sites. EMR is the average number of polymorphic bands [48].

### 4.5. Population Genetic Structure Analysis

Four hundred and ten bands were selected from 453 bands after deleting low frequency bands (minor allele frequency (MAF ≤ 5% and MAF ≥ 95%) for further structure, linkage disequilibrium (LD), and association analyses. The population genetic structure was analyzed using STRUCTURE V2.3.4 software (http://pritchardlab.stanford.edu/structure.html) [33]. The pre-defined K (number of groups in a population) value from 1 to 11 using admixture models was set to run STRUCTURE 10 times, with a burn-in of 10,000 and 100,000 iterations of Markov chain convergence for each run. A K value was chosen once the estimate of lnPr(X|K) peaked in the range of 1–11 subpopulations. delta K (ΔK), which is an ad hoc quantity correlated to the second order change in the log probability of data with relation to the number of clusters, was detected to the most probable value of k by the model choice criterion [49]. The maximum likelihood in the run was conducted to subdivide the varieties into different subgroups by using the maximum membership probability and a membership probability threshold of 0.60 among subgroups, and the varieties below 0.60 were retained in the admixed group (AD).

### 4.6. Evaluation of Linkage Disequilibrium

The squared band-frequency correlations (r^2^) between all combinations of marker bands (410) were used to evaluate the significance of pairwise LD using TASSEL version 2.1 (http://sourceforge.net/projects/tassel/) [50] with 1000 permutations. Each pair of bands were detected to have significant LD if *p* < 0.01.

### 4.7. Association Analysis

The associations of EST-SSR and SCoT markers with rust traits over two years in the presence of population structure were investigated using Tassel software (TASSEL 2.1), which is based on adopting a general linear model (GLM) [50]. The population structure was considered in the association analysis of all phenotypic traits. Based on the Q model, the GLM pattern in the TASSEL software was applied to do multiple tests of significant associations between detected bands and rust phenotypes.

## 5. Conclusions

This study illustrates a high level of genetic diversity in orchardgrass and it was found that genetic variance mainly exists within populations. Comparison with MI between EST-SSRs and SCoTs shows that SCoTs have higher marker efficiency than EST-SSRs. The UPGMA cluster and STRUCTURE analyses show that these results are both correlated with geographic distribution. LD results indicate that a high extent of LD is present in the orchardgrass accessions. In the association analysis, 20 band panels that associate with rust trait in both 2011 and 2012 were detected, and these bands may apply to MAS that select rust resistant lineages.

## Supplementary Materials

Supplementary materials can be accessed at: http://www.mdpi.com/1420-3049/21/1/66/s1.
