# Supplementary material for: Genetic Diversity and Association of EST-SSR and SCoT Markers with Rust Traits in Orchardgrass (Dactylis glomerata L.)
Source: Molecules. 2016 Jan 8;21(1):66. doi: 10.3390/molecules21010066 (PMC6273750; doi:10.3390/molecules21010066)
Supplement: Supplementary file 1 [file molecules-21-00066-s001.pdf]

Supplementary Materials: Genetic Diversity and Association of EST-SSR and SCoT Markers with Rust Traits in Orchardgrass (*Dactylis glomerata* L.)

Haidong Yan, Yu Zhang, Bing Zeng, Guohua Yin, Xinquan Zhang, Yang Ji, Linkai Huang, Xiaomei Jiang, Xinchun Liu, Yan Peng, Xiao Ma and Yanhong Yan

Table S1. The primer information for EST-SSR primers in orchardgrass.

Forward Primer Sequence		Reverse Primer Sequence	
H37-cnl35F	AAGTGAGCACAACGACACGA	H38-cnl35R	CGATCCAAAGAAGCAAAGATG
H39-cnl37F	CTGCCTCGCGTGAAAGATA	H40-cnl37R	CCTCCTCGATCTGGATGGT
H41-cnl39F	TACCTGTGCGGCGATGAAT	H42-cnl39R	CAGGAGCAGGAGAACGTGAA
H47-cnl51F	CTAGGGTTTCCCACCTCTCA	H48-cnl51R	AATGTCCTTGGCGTTGCT
H49-cnl53F	CGCAGCAAGTAGGGTTAGGA	H50-cnl53R	CCTCGTGGTGGATCTGCAT
H53-cnl61F	CACGAGTGCAGAGCTAGACG	H54-cnl61R	ACAACAACCCGACTGCTACC
H55-cnl74F	GGCTCGAGCTTAAAACCCTA	H56-cnl74R	CTCCATCCATTCTTGCCATCT
H59-cnl100F	CGTCGTCCTCTGCTGTGAG	H60-cnl100R	AGGTCGTCCATCTGCTGCT
H61-cnl101F	GCGGAGGAGAGAAAGCAAT	H62-cnl101R	AGGTCGTCCATCTGCTGCT
H63-cnl107F	CCACCAGCTTCCTTCTCTTG	H64-cnl107R	CCCTCAACAAATCGAACTCC
H67-cnl115F	CGAGAAGAAGGTGGTGTCGT	H68-cnl115R	AGGTCGTGGAAGGTCTTGG
H69--cnl119F	ATCGTCTCCTCCTCCTCCA	H70-cnl119R	ATGCCTCGGTGGACTGGTA
H71--cnl130F	AAATGTTGAGCAACGGGAGCT	H72-cnl130R	ACTTCATAGGGCGGAGGTCT
H75-cnl144F	AGAAGGCGGCTCAGAAGAAG	H76-cnl144R	GCTCCAACTCAGAATCAACAA
H77-cnl147F	GGCTAGGGTTTCGACTCCTC	H78-cnl147R	AGATGGCGAACTCGACCTG
H79-cnl152F	ACAAAGGCTCACCGTGGAA	H80-cnl152R	GTCGGAGGCGATGAACTCT
H81-cnl156F	CTTCCCTCACCTCAAGCAGT	H82-cnl156R	GACAGCAGCACACCGAGTT
H83-cnl158F	CTCATCCCACCACCACCAC	H84-cnl158R	CCCTGAAGAAGTCGAACACG

Table S2. The band information of EST-SSRs in orchardgrass.

Primer Pairs Bands	Total Number of Bands	Number of Polymorphic Bands	Percentage of Polymorphic Bands (%)
H037.038	12	11	91.67
H039.040	10	10	100.00
H041.042	8	7	87.50
H047.048	8	7	87.50
H049.050	6	5	83.33
H053.054	7	7	100.00
H055.056	6	6	100.00
H059.060	9	8	88.89
H061.062	7	6	85.71
H063.064	11	11	100.00
H067.068	11	9	81.81
H069.070	14	12	85.71
H071.072	9	9	88.89
H075.076	8	7	87.50
H077.078	10	10	100.00
H079.080	10	9	90.00
H081.082	11	9	81.81
H083.084	7	5	71.43
Total	164	148	90.24

Table S3. The information about SCoT primers in orchardgrass.

Primer Name	Sequences(5′-3′)
SCoT1	CAACAATGGCTACCACCA
SCoT2	CAACAATGGCTACCACCC
SCoT5	CAACAATGGCTACCACGA
SCoT6	CAACAATGGCTACCACGC
SCoT8	CAACAATGGCTACCACGT
SCoT10	CAACAATGGCTACCAGCC
SCoT14	ACGACATGGCGACCACGC
SCoT16	ACCATGGCTACCACCGAC
SCoT25	ACCATGGCTACCACCGGG
SCoT26	ACCATGGCTACCACCGTC
SCoT27	ACCATGGCTACCACCGTG
SCoT34	ACCATGGCTACCACCGCA
SCoT35	CATGGCTACCACCGGCCC
SCoT36	GCAACAATGGCTACCACC
SCoT37	CAATGGCTACCACTAGCC
SCoT38	CAATGGCTACCACTAACG
SCoT39	CAATGGCTACCACTAGCG
SCoT40	CAATGGCTACCACTACAG
SCoT41	CAATGGCTACCACTGACA
SCoT42	CAATGGCTACCATTAGCG
SCoT44	CAATGGCTACCATTAGCC
SCoT45	ACAATGGCTACCACTGAC

Table S4. The band information of SCoTs in orchardgrass.

Primer Pairs Bands	Total Number of Bands	Number of Polymorphic Bands	Percentage of Polymorphic Bands (%)
SCoT1	14	9	64.29
SCoT2	17	17	100.00
SCoT5	10	10	100.00
SCoT6	10	7	70.00
SCoT8	20	20	100.00
SCoT10	19	17	89.47
SCoT14	19	18	94.74
SCoT16	10	10	100.00
SCoT25	14	14	100.00
SCoT26	12	9	75.00
SCoT27	10	9	90.00
SCoT34	13	13	100.00
SCoT35	9	8	88.89
SCoT36	8	8	100.00
SCoT37	14	14	100.00
SCoT38	13	13	100.00
SCoT39	17	16	100.00
SCoT40	15	15	100.00
SCoT41	12	12	100.00
SCoT42	20	20	100.00
SCoT44	13	13	100.00
Total	289	272	94.12

Table S5. The detailed information about accession code, name, origin, and rust resistance score in orchardgrass.

Accession Code	Accession Name	Origin	Ploidy	Rust Resistance Score in Different Year	
				2011	2012
1	98-102	United States	Tetraploid	6	5
2	1473	Australia	Tetraploid	6	5
3	2122	Animal Husbandry Research Institute, Hubei, China	Tetraploid	6	6
4	947	Japan	Tetraploid	6	5
5	02-114	Qujing, Yunnan, China	Tetraploid	6	6
6	02-115	Deqin, Yunnan, China	Tetraploid	6	5
7	1436	Jiangshu, China	Tetraploid	6	5
8	1824	Animal Husbandry Research Institute, Chinese Academy of Agricultural Sciences, Beijing, China	Tetraploid	6	6
9	1993	Animal Husbandry Research Institute, Chinese Academy of Agricultural Sciences, Beijing, China	Tetraploid	6	5
10	PI610830	Spain	Tetraploid	6	6
11	PI610822	Spain	Tetraploid	6	6
12	PI173693	Turkey	Tetraploid	6	5
13	PI237590	Israel	Tetraploid	6	5
14	PI578587	Portugal	Tetraploid	5	5
15	PI325293	Russian Federation	Tetraploid	6	6
16	PI312450	Former Soviet Union	Tetraploid	6	5
17	PI418672	Italy	Tetraploid	5	5
18	PI305497	Poland	Tetraploid	5	5
19	PI384018	Poland	Tetraploid	6	6
20	PI399466	Finland	Tetraploid	5	6
21	PI237268	Finland	Tetraploid	5	5
22	PI598424	Bulgaria	Tetraploid	5	6
23	PI598423	Ireland	Tetraploid	6	5
24	PI231613	Iran	Tetraploid	5	5
25	PI380812	Iran	Tetraploid	5	5
26	PI308794	India	Tetraploid	6	6
27	PI269885	Pakistan	Tetraploid	6	5
28	PI237586	Tunisia	Tetraploid	5	6
29	PI578667	Egypt	Tetraploid	6	5
30	PI231727	Chile	Tetraploid	5	5
31	PI469234	Australia	Tetraploid	6	5
32	2410	Xinjiang, China	Tetraploid	1	1
33	79-118	Netherlands	Tetraploid	1	1
34	2681	Canada	Tetraploid	1	2
35	Anba	Denmark	Tetraploid	2	2
36	2106	Xinjiang, China	Tetraploid	1	2
37	02-105	Dazhou, China	Tetraploid	1	2
38	02-106	Baoxing, China	Tetraploid	1	2
39	90-130	Maoxian, Sichuan, China	Tetraploid	1	1
40	1819	Animal Husbandry Research Institute, Chinese Academy of Agricultural Sciences, Beijing, China	Tetraploid	1	1
41	Kaimo	Xinjiang, China	Tetraploid	2	2
42	PI231517	Morocco	Tetraploid	1	2
43	PI265567	France	Tetraploid	1	2
44	PI277836	Turkey	Tetraploid	1	2
45	PI249732	Greece	Tetraploid	2	1
46	PI231535	Portugal	Tetraploid	2	1
47	PI632497	Tunisia	Tetraploid	1	2
48	PI287804	Spain	Tetraploid	1	1
49	PI302892	Spain	Tetraploid	1	2
50	PI292587	Israel	Tetraploid	1	1
51	PI235281	France	Tetraploid	2	1
52	PI578591	Portugal	Tetraploid	1	1

Table S5. *Cont.*

Accession Code	Accession Name	Origin	Ploidy	Rust Resistance Score in Different Year	
				**2011**	**2012**
53	PI111536	Russian Federation	Tetraploid	2	2
54	PI345603	Former Soviet Union	Tetraploid	1	2
55	PI311042	Romania	Tetraploid	1	1
56	PI250960	Serbia and Montenegro	Tetraploid	2	2
57	PI231469	Greece	Tetraploid	1	1
58	PI288997	Libya	Tetraploid	2	2
59	PI289003	Hungary	Tetraploid	2	2
60	PI418667	Belgium	Tetraploid	2	2
61	PI237175	Netherlands	Tetraploid	2	1
62	PI196419	Denmark	Tetraploid	2	2
63	PI634311	Ireland	Tetraploid	2	2
64	PI273738	Argentina	Tetraploid	2	2
65	PI250928	Iran	Tetraploid	1	2
66	PI237585	India	Tetraploid	2	1
67	PI419517	Japan	Tetraploid	2	1
68	PI595173	Xinjiang, China	Tetraploid	2	1
69	PI632580	Afghanistan	Tetraploid	2	2
70	PI231480	Algeria	Tetraploid	2	2
71	PI578557	United states	Tetraploid	2	1
72	PI578661	Colombia	Tetraploid	2	1
73	PI285099	Australia	Tetraploid	2	2
74	PI578634	Morocco	Tetraploid	2	1
75	PI578635	Morocco	Tetraploid	1	1
